# Reversal of Bioprosthetic Aortic Valve Thrombosis Using Rivaroxaban—A Case Report

**DOI:** 10.3389/fcvm.2020.00087

**Published:** 2020-05-27

**Authors:** Harish Sharma, Vincenzo Vetrugno, Peter Ludman

**Affiliations:** ^1^Institute of Cardiovascular Sciences, University of Birmingham, Birmingham, United Kingdom; ^2^Department of Cardiology, Queen Elizabeth Hospital, University Hospitals Birmingham NHS Foundation Trust, Birmingham, United Kingdom; ^3^Department of Cardiovascular and Thoracic Sciences, Fondazione Policlinico Universitario A. Gemelli IRCCS, Catholic University of the Sacred Heart, Rome, Italy

**Keywords:** rivaroxaban, valve thrombosis, thrombosis reversal, Bioprosthetic aortic valve, NOAC

## Abstract

**Background:** Bioprosthetic valve thrombosis (BPVT) is a rare but recognized complication causing valve dysfunction. In subacute valve thrombosis, systemic oral anticoagulation is recommended. However, there is little data comparing the efficacy of warfarin and novel oral anticoagulant (NOAC) therapy in this setting.

**Case Summary:** A patient developed subacute BPVT 11 years post-implantation. The patient was initially treated with warfarin for a period of 6 months, with limited effect. Following replacement of warfarin with rivaroxaban, there was significant reversal of the BPVT, as represented by a reduction in transaortic maximal velocity (Vmax) from 4.1 to 3 m/s over 7 months.

**Discussion:** Systemic oral anticoagulation can be an effective treatment for subacute valve thrombosis. Guidelines currently recommend warfarin as first line but NOACs can be considered in such patients and may be more effective than warfarin. Randomized controlled trials are required to further establish the optimal anticoagulation for patients with subacute BPVT.

## Introduction

Bioprosthetic valve thrombosis (BPVT) is a rare but recognized complication of prosthetic valve replacements and can contribute to significant valve dysfunction and clinical deterioration. Acute valve thrombosis can be life-threatening emergency, while sub-acute BPVT may present insidiously. According to European Society of Cardiology (ESC) guidelines, anticoagulation using a vitamin K antagonist (VKA) and/or unfractionated heparin is the first-line treatment of BPVT (class I, level of evidence C) (1).

Novel oral anticoagulants (NOACs) have emerged as an effective alternative to VKA therapy and have been shown to have a safer risk profile compared to warfarin in patients with atrial fibrillation (AF) (2, 3). Despite being increasingly prescribed in a variety of clinical settings, the use of NOACs in the treatment of BPVT have not been extensively studied and there is a paucity of guidelines pertaining to their role in this setting (1).

## Case

An 89-year-old man presented to the Cardiology clinic for routine follow up. In 2007, he had been diagnosed with severe degenerative aortic and mitral valve regurgitation necessitating surgery. Aortic and mitral valve replacements were performed with a 25 mm Edwards bioprosthesis and a 31 mm Carpentier-Edwards Perimount bioprosthesis, respectively. The patient also had a prior history of paroxysmal atrial fibrillation (AF) and therefore radiofrequency ablation and left atrial appendage exclusion was also performed and the patient discharged on warfarin.

In 2013, the patient was re-admitted to hospital with heart failure. A transthoracic echocardiogram (TTE) revealed severe mitral regurgitation, moderate mitral stenosis and tricuspid regurgitation. Re-do tissue mitral valve replacement (27 mm Perimount) and tricuspid repair (34 mm Edwards MC3 annuloplasty ring) surgery was performed successfully. The patient remained on warfarin for AF stroke prophylaxis until 6 months post-operatively, when he developed frank haematuria following a transurethral resection of the prostate procedure. The warfarin was stopped after careful consideration of the risks of bleeding vs. the risk of AF-related embolic stroke. Annual 24-h ambulatory ECG monitoring in 2015 and 2016 revealed only sinus rhythm with no evidence of a recurrence of AF.

**Figure 1:**
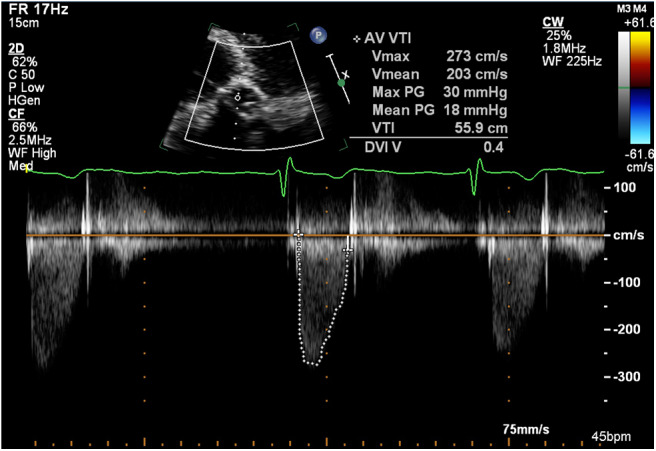
Transthoracic echocardiogram (January 2017): Continuous wave doppler in the apical 5 chamber view demonstrating a maximum transaortic velocity of 2.7 m/s.

In 2017, a routine surveillance TTE demonstrated stable valve prostheses and a transaortic maximum velocity (Vmax) of 2.7 m/s (Figure 1).

**Figure 2:**
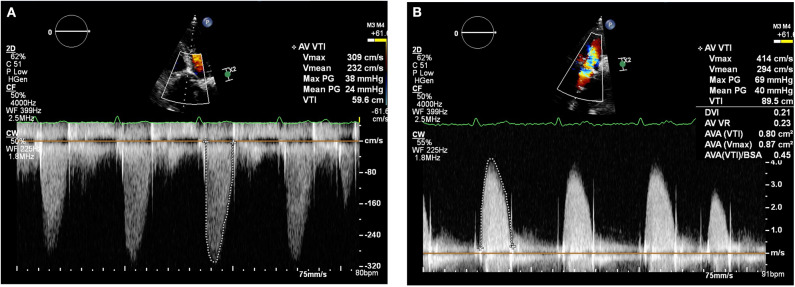
Transthoracic echocardiogram (January 2018): Continuous wave doppler in the apical 5 chamber view **(A)** and right parasternal long axis view **(B)** demonstrating a maximum transaortic velocity of 4.1 m/s.

By January 2018 however, the patient developed mild breathlessness and a repeat TTE revealed an elevated Vmax of 4.1 m/s (Figure 2) suggestive of a significantly elevated transvalvular pressure gradient. The prosthetic valve leaflets appeared thickened and calcified with reduced excursion. The most likely diagnosis at this stage was irreversible bioprosthetic valve degeneration, which can be expected approximately 10 years following implantation. A rare differential diagnosis was subacute reversible valve thrombosis. The patient was still able to exercise in the gym and on the basis that his dyspnoea was not limiting, the decision was made to monitor his symptoms 6 monthly, with a plan to consider valve-in-valve transcatheter aortic valve intervention (TAVI) if his symptoms worsened.

**Figure 3:**
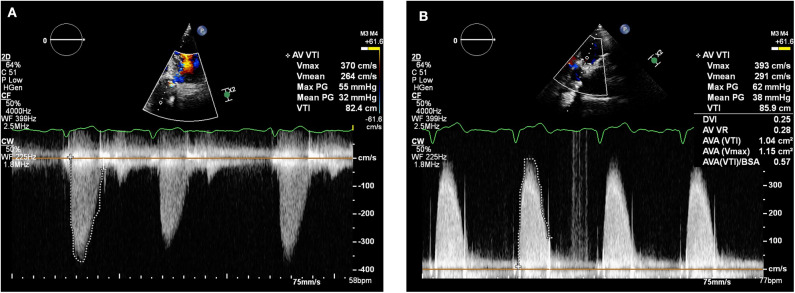
Transthoracic echocardiogram (August 2018): Continuous wave doppler in the apical 5 chamber view **(A)** and right parasternal long axis view **(B)** demonstrating a maximum transaortic velocity of 3.9 m/s.

In February 2018, a holter monitor revealed atrial flutter throughout and so anticoagulation was recommended for stroke prophylaxis. The patient's general practitioner commenced warfarin and after a 6-month period, repeat TTE in August 2018 revealed slight improvement in the transaortic Vmax which reduced to 3.9 m/s (Figure 3). Improvement in the transvalvular gradient raised the suspicion that the valve may be thrombosed, rather than degenerative.

**Figure 4:**
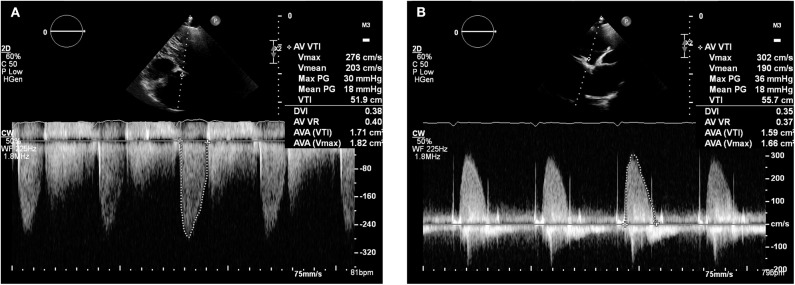
Transthoracic echocardiogram (March 2019): Continuous wave doppler in the apical 5 chamber view **(A)** and right parasternal long axis view **(B)** demonstrating a maximum transaortic velocity of 3.0 m/s.

As the patient's dyspnoea remained mild, a watchful waiting policy was adopted for the aortic stenosis. The only change to his management was to replace his warfarin with rivaroxaban, as per latest guidelines for stroke prophylaxis (2). After 7 months, a subsequent TTE in March 2019 showed the transaortic velocity to have reduced to 3 m/s giving a mean transvalvular gradient of only 18 mmHg (Figure 4) compared to a previous mean gradient of 40 mmHg in January 2018. This suggests significant improvement of the aortic valve stenosis from severe to only mild (Table 1). The degree of reversal of the transvalvular velocity following rivaroxaban strongly suggests that valve thrombosis was the cause of the bioprosthetic aortic valve re-stenosis, rather than bioprosthetic valve degeneration which would not improve to this extent following anticoagulation. As the diagnosis was clear from the significant improvement in valve parameters on TTE, further imaging (which would have definitively proven the diagnosis) was not performed as it would not have changed the course of management for the patient. Nevertheless, this case demonstrates that significant improvement of presumed subacute bioprosthetic valve thrombosis can be achieved with NOACs.

**Table 1:** Demonstrating the change in echocardiographic parameters over time.

	**Post-op (2013)**	**January 2017**	**January 2018**	**August 2018**	**March 2019**
Peak gradient	21 mmHg	30 mmHg	69 mmHg	62 mmHg	34 mmHg
Mean gradient	13 mmHg	16 mmHg	40 mmHg	38 mmHg	19 mmHg
Transaortic Vmax	2.3 m/s	2.7 m/s	4.1 m/s	3.9 m/s	3.0 m/s
EOAi	1.08 cm^2^/m^2^	1.02 cm^2^/m^2^	0.45 cm^2^/m^2^	0.58 cm^2^/m^2^	0.87 cm^2^/m^2^
Anticoagulation	None	None	Warfarin	Rivaroxaban	Rivaroxaban

## Timeline


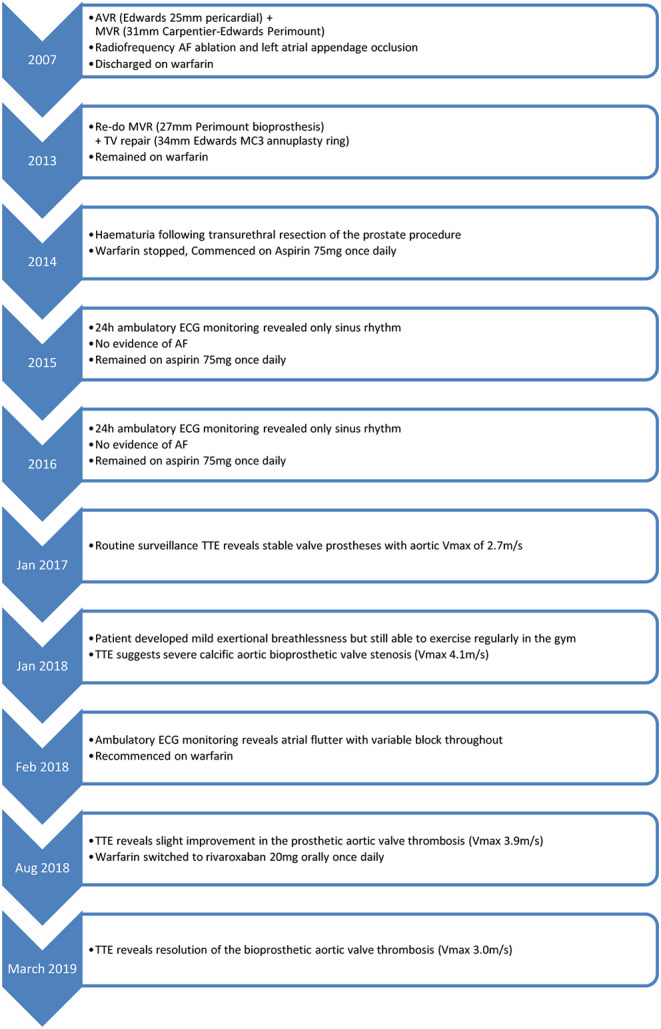


## Decision Making Process


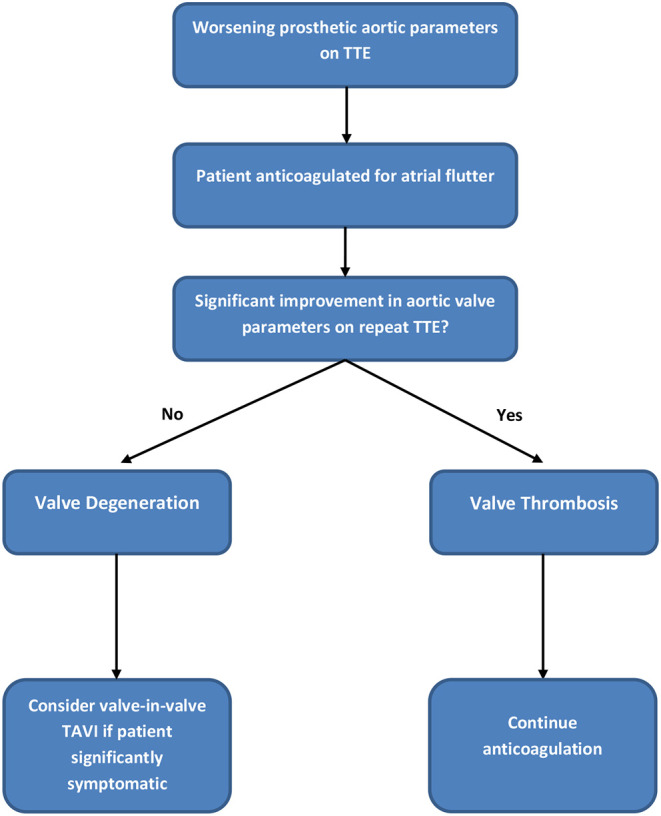


## Follow-Up and Outcome

The patient will continue to be monitored with annual surveillance of the valve by transthoracic echocardiography. At the time of writing, the patient has not undergone further echocardiography, but his symptoms were reviewed in February 2020 and he no longer reported any dyspnoea.

## Limitations

While this case demonstrates the improvement of bioprosthetic aortic valve parameters following anticoagulation with rivaroxaban, this case represents only one patient and further clinical studies are required to determine the efficacy of NOACs in the treatment of BPVT. While the period of treatment on warfarin did not significantly alter the bioprosthetic aortic valve parameters, it is not possible to be certain that the warfarin was fully therapeutic during this treatment period, as the international normalized ratio (INR) values are not available to us. However, United Kingdom patients commenced on warfarin are under close supervision of an outpatient nurse-led anticoagulation service which closely monitors INR values and advises dose adjustments. Physicians are informed if their patients are unable to maintain therapeutic INR levels. As we were not informed of any issues regarding this patient's INR levels, we assume that the warfarin was in the therapeutic range during the 6-month period that he was treated with warfarin.

## Discussion

In this case, the treatment of atrial flutter with anticoagulation resulted in inadvertent unmasking of subacute aortic BPVT, for which the anticoagulation (particularly rivaroxaban) was an effective treatment. Systemic anticoagulation is effective in preventing mechanical valve thrombosis, but the role of anticoagulation in the prophylaxis and management of bioprosthetic valve thrombosis (BPVT) is less understood. International guidelines differ in their recommendations for the requirement and/or duration of anticoagulation following surgical bioprosthetic valve implantation (1, 4). Despite these recommendations, BPVT may be indolent (5) or occur late after valve implantation. In one case series, 35% occurred within 12 months of initial implantation and median time-to-explantation was 24 months. Less than 11% occurred after 10 years (6). In the case described here, the presentation was very late after implantation (11 years) and subacute (mild dyspnoea only). Nevertheless, BPVT should be considered as an important differential of valve deterioration owing to the reversible nature of the condition.

The patient described in this case developed atrial flutter requiring anticoagulation. Many patients with longstanding bioprosthetic valve replacements have indications for anticoagulation. In this circumstance, the 2017 European guidelines state that vitamin K antagonists should be preferred, but NOACs can be also be used from the third month onwards (1). The choice of anticoagulant is particularly important in this cohort of patients, as suboptimal anticoagulation has been shown to increase risk of BPVT (6).

Much of the recent evidence of valve thrombosis treatment comes from studies assessing patients following TAVI in which subclinical thrombosis is more common. In these trials and registries, markers of subclinical valve thrombosis such as reduced leaflet mobility and hypoattenuated leaflet thickening were identified on computer tomography (CT) scans. Anticoagulation with warfarin compared to dual antiplatelet therapy was found to reduce the incidence of subclinical valve thrombosis (7). Other case series have corroborated that anticoagulation is successful at restoring TAVI function following subclinical thrombosis (8).

A recent retrospective case series compared the outcomes of patients with BPVT treated with warfarin (*n* = 15) vs. surgery and/or thrombolysis (*n* = 17). As both treatment strategies reduced the mean transvalvular gradient to a similar extent, anticoagulation was considered the best first-line treatment in a haemodynamically stable patient because of lower risk (9). It remains uncertain whether the optimal anticoagulant is a member of the vitamin K antagonist or NOAC family and to date, there have been no clinical trials comparing the efficacy of warfarin with a NOAC in the treatment of BPVT.

Interestingly, although rivaroxaban appears to have been effective in the *treatment* of BPVT in this case report, it has not been effective in *preventing* BPVT in other studies. The recent GALILEO randomized controlled trial comparing rivaroxaban vs. antiplatelet therapy for the prevention of TAVI thrombosis was terminated early due to an increase in all-cause mortality, thromboembolic events and bleeding in the rivaroxaban arm (10). Nevertheless, treatment of an established valvular thrombus is a different clinical scenario than the prevention of thrombus in an otherwise well-functioning valve. This case report demonstrates in one patient that significant improvement of transaortic forward flow velocity can be achieved in presumed subacute BPVT treated with a NOAC. This may represent a potential role for NOACs particularly if VKA therapy is ineffective or not tolerated. Randomized control trials are required to further clarify the role of NOACs in the treatment of BPVT.

## Data Availability Statement

All datasets generated for this study are included in the article/supplementary material.

## Ethics Statement

Ethical review and approval was not required for the study on human participants in accordance with the local legislation and institutional requirements. The patients/participants provided their written informed consent to participate in this study.

## Author's Note

This paper was the original work of the authors who have all seen and approved of the paper and authorship. The article has not been published elsewhere and is not under consideration in any other journals.

## Author Contributions

All authors listed have made a substantial, direct and intellectual contribution to the work, and approved it for publication.

## Conflict of Interest

The authors declare that the research was conducted in the absence of any commercial or financial relationships that could be construed as a potential conflict of interest.
